# Expression dynamics of miRNAs and their targets in seed germination conditions reveals miRNA-ta-siRNA crosstalk as regulator of seed germination

**DOI:** 10.1038/s41598-017-18823-8

**Published:** 2018-01-19

**Authors:** Shabari Sarkar Das, Sandeep Yadav, Archita Singh, Vibhav Gautam, Ananda K. Sarkar, Asis K. Nandi, Prakash Karmakar, Manoj Majee, Neeti Sanan-Mishra

**Affiliations:** 1 Plant RNAi Biology Group, International Centre for Genetic Engineering and Biotechnology, Arina Asaf Ali Marg, New Delhi, 110067 India; 2 National Institute of Plant Genome Research, Aruna Asaf Ali marg, New Delhi, 110067 India; 3 Department of Botany and Forestry, Vidyasagar University, Midnapore, West Bengal India

## Abstract

Seed germination paves the way for the dormant embryo to establish itself as a new plant marking the first critical step in postembryonic plant growth and development. Germination starts with the uptake of water (imbibition), followed by induction of transcription, translation, energy metabolism, and cell division processes. Although small RNAs have been implicated in many developmental processes, their role during seed germination stages and conditions remained elusive. Here we show that seed germination conditions, like imbibition and temperature, dynamically regulate the expression of many developmentally important miRNAs and their targets. We have identified 58 miRNAs belonging to 30 different families at different seed germination conditions. Amongst these, 15 miRNAs and their targets were significantly differentially expressed in *Arabidopsis* seeds in dry and 12 h, 24 h and 48 h of imbibition. Interestingly, differential expression of miR390, which targets trans-acting siRNA locus (*TAS3*) derived transcripts, resulted in alteration of tasiR-*ARF* mediated regulation of expression of target *AUXIN RESPONSE FACTORs (ARF2/3/4*). Our results suggest that the dynamic expression of several miRNAs, their targets, and a crosstalk between miRNA and ta-siRNA pathways contribute to the regulation of seed germination in *Arabidopsis thaliana*.

## Introduction

Seed germination process, which marks the transition from seed to seedling stage, plays a crucial role in the complete life cycle of higher plants. Dormant seeds germinate when the environmental conditions, such as temperature, water or humidity are favourable^1^. The contrasting physiological event of seed germination is dormancy, which is regarded as the temporary failure or block of a viable seed to complete germination under seemingly unfavourable conditions and is an adaptive feature for optimizing the timing of germination^2^. Seed germination starts with the uptake of water (imbibition) by the quiescent dry seed and is considered as complete when radicle emerges out from the seed coat. A complex co-ordination of different molecular, physiological and environmental factors govern the dynamic and triphasic process of seed germination^2,3^.

Small non-coding RNAs (of 19–24 nucleotides length) play diverse roles in growth, development, morphogenesis, and stress responses in both plants and animals^4–8^. There are two major classes of small non-coding RNAs - short interfering RNAs (siRNAs) and microRNAs (miRNAs) that regulate their target genes by binding to the complementary sequences and generally by making a cleavage in the target mRNA^9,10^ or by blocking their translation^10–12^. Recently, trans-acting small interfering RNAs (ta-siRNA) have also been implicated in plant development (Axtell, 2013). ta-siRNAs belong to a plant-specific class of endogenous small RNAs, whose biogenesis requires an initial process of specific miRNA-mediated cleavage of their precursors^2,13^. The functions of miRNAs are also regulated by several hormones and stresses^14–16^. Recently, it has been shown that proteins involved in small RNA biogenesis such as DICER LIKE1 (DCL1) plays significant role in embryogenesis and seed development in *Arabidopsis*^1^. This indicates involvement of small RNAs in seed development. Mutation in *ARF10*, which is the target of miR160, results in developmental defects in seeds through induced expression of ABA responsive genes^15^. miR159 regulates floral development, fertility and seed germination by targeting and negatively regulating *MYB* transcript level^16^; miR417 negatively regulate seed germination under salt stress condition and over expression of miR402 enhances the seed germination under stress conditions in *Arabidopsis thaliana*^17,18^.

Previous study reported 115 known miRNAs and 167 novel miRNAs in maize seeds imbibed for 24 h, which is very early stages of seed germination^19^. They identified 24 conserved miRNA families in both dry and imbibed maize seeds through deep sequencing. Few novel and known miRNAs and some of their targets were validated^20^. Deep sequencing of small RNAs from rice seed embryos identified 59 known and 230 novel miRNAs differentially regulated in the early stages of seed germination^21^. Moreover, in eudicot (*Nelumbo nucifera)* seed germination, 145 known and 78 novel miRNAs were identified^22^. These reports indicate the association of more number of small RNAs in the dynamic seed germination procedure. However, little is known about the condition specific regulation of small RNAs and their targets, which are potentially important contributors to the early stages of *Arabidopsis* seed germination.

In this study, we have identified both conserved and non-conserved small RNAs in both dry and imbibed *Arabidopsis* seeds under stratified (4 °C) and non-stratified conditions. We used miRNA- microarray approach to identify miRNAs functionally relevant to early stages of seed germination. We validated the expression of 15 such miRNAs and their targets at 12 h, 24 h and 48 h imbibition at 4 °C and room temperatures. Besides this, the expression level of miR390 was significantly high at both 24 h/RT and 24 h/4 °C conditions, which is required for processing of *TAS3* transcripts^23^. miR390 is required for the processing of *TAS3* transcript into functional tasiR-*ARF* that targets and negatively regulate the *ARF* family members *ARF2, ARF3*, and *ARF4*^14,24^. The correlation of altered expression of miR390 and *ARF2/3/4* indicate that a crosstalk of both miRNAs, ta-siRNAs, and their targets contributes to the dynamic seed germination process.

## Results

### Expression of miRNAs are differentially regulated in seeds under germination conditions

**Figure 1:**
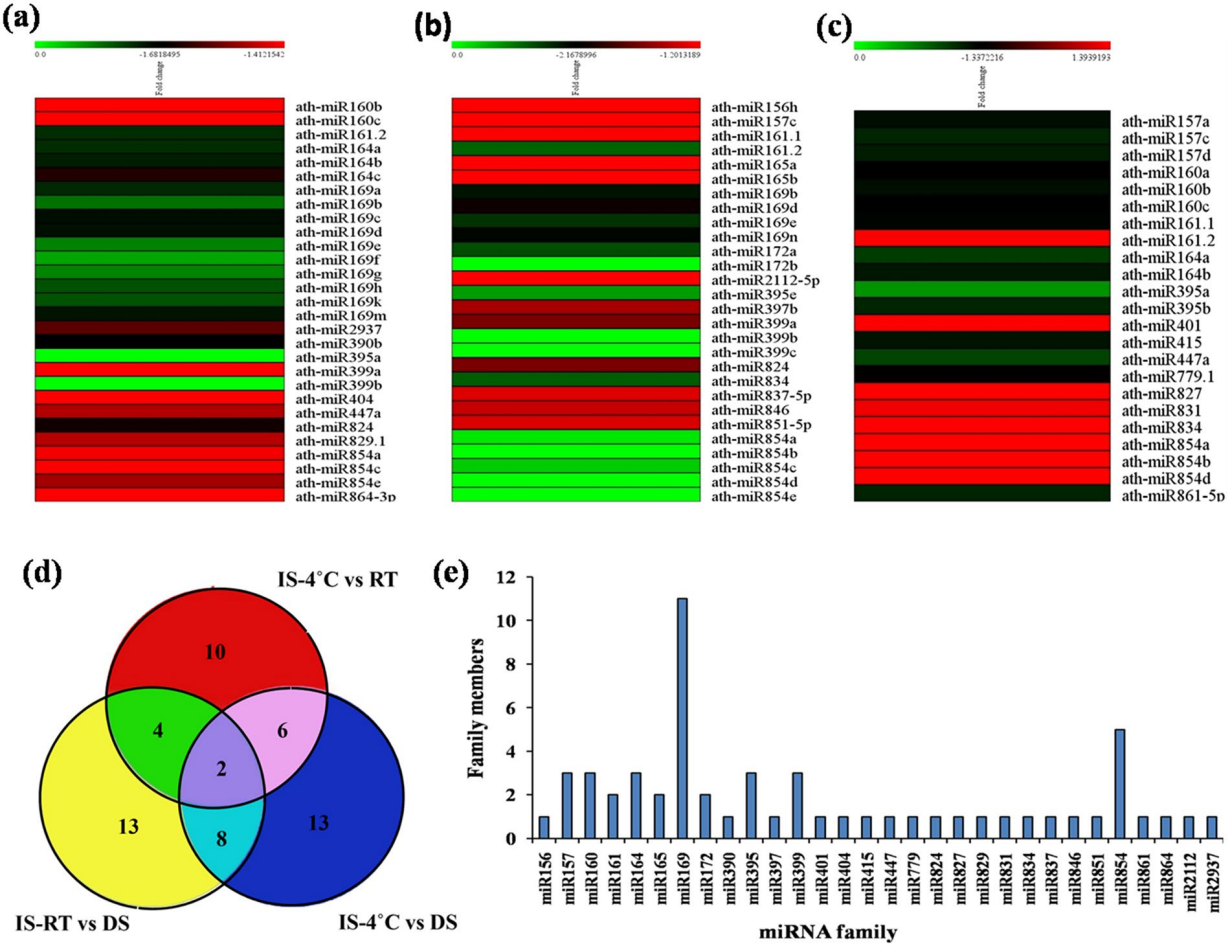
Microarray based expression patterns of miRNAs at different conditions of seed germination in *Arabidopsis thaliana*. (**a)** Heat map at cold imbibitions (4 °C) vs. dry seeds (DS). **(b)** Heat map at room temperature (RT) vs. dry seed (DS). **(c)** Heat map at cold imbibitions (4 °C) vs. room temperature (RT) [For generating heat map we used MeV(Multiple Experiment Viewer) (http://mev.tm4.org/)]. The bars represent the scale of expression levels of the miRNAs. During microarray, we have pooled all the cold imbibed (IS-4 °C) and room temp imbibed (IS-RT) total RNAs separately, and the microarray was performed with two biological replicates for each individual samples. **(d)** The Venn diagram represents the comparison of the known miRNAs between three different conditions (DS, IS-4 °C and IS-RT) used in the microarray experiment. **(e)** The graph represents the miRNA families and their respective family members that were detected in the microarray analysis.

In order to identify miRNAs that are involved in seed germination process in *Arabidopsis*, we used miRNA microarray approach. Microarray was performed using total RNA from imbibed seeds (IS) at room temperature (RT), imbibed seeds at 4 °C and dry seeds (DS), and a three way comparison was made to identify differently expressed miRNAs. We identified a total of 58 miRNAs differently expressed in (i) IS-4 °C vs. DS (Fig. 1a), (ii) IS-RT vs. DS (Fig. 1b) and lastly, (iii) IS-4 °C vs. IS-RT (Fig. 1c). We found that specific miRNAs were upregulated and down regulated in each of the three cases. The results identified 29 miRNA genes in IS- 4 °C vs. DS (Fig. 1a), 28 miRNA genes in IS-RT vs. DS (Fig. 1b) and 23 miRNA genes in IS-4 °C vs. IS-RT (Fig. 1c). Among the 29 miRNAs in IS- 4 °C vs. DS (Fig. 1a), only two miRNAs were upregulated and rest were downregulated. In case of 28 miRNAs in IS-RT vs. DS (Fig. 1b), five miRNAs were upregulated and rest were downregulated, whereas among 23 miRNAs in IS-4 °C vs. IS-RT (Fig. 1c), only eight miRNA genes were upregulated and rest were downregulated.

**Figure 2:**
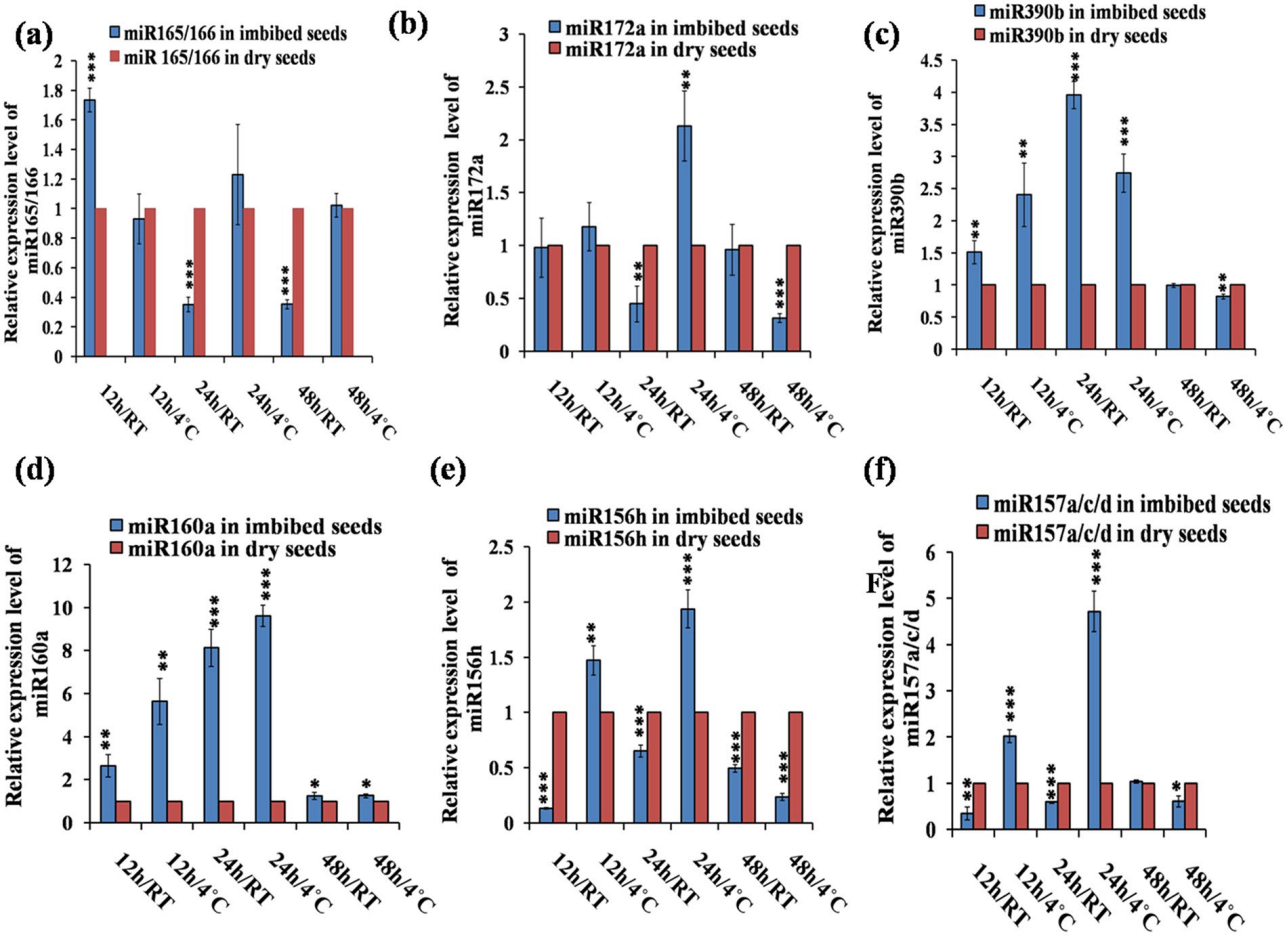
Quantitative RT-PCR based validation of expression of mature miRNAs in imbibed seeds, in comparison to dry seeds, in *Arabidopsis thaliana*. (The expression profiles obtained from qRT-PCR slightly varied with the microarray based miRNA expressions, probably due to the pulling of total RNAs during microarray). The qRT-PCR validation of miRNAs were done at six different germination stages as 12 h/RT, 12 h/4 °C, 24 h/RT, 24 h/4 °C, 48 h/RT and 48 h/4 °C each compared to dry seed. The expression values presented here were the means of three biological replicates ± standard deviation (SD). The *Arabidopsis ACTIN7* was used for each samples as an endogenous control. (**a**) ath-miR165/166; (**b**) ath-miR172a; (**c**) ath-miR390b; (**d**) ath-miR160a; (**e**) ath-miR156h; (**f**) ath-miR157a/or, c/or, d. In *Arabidopsis*, miR157a, c and d have the same mature sequence. Asterisks indicate significant statistical differences: ***P < 0.001, **P < 0.01, *P < 0.05 (One-way ANOVA).

**Figure 3:**
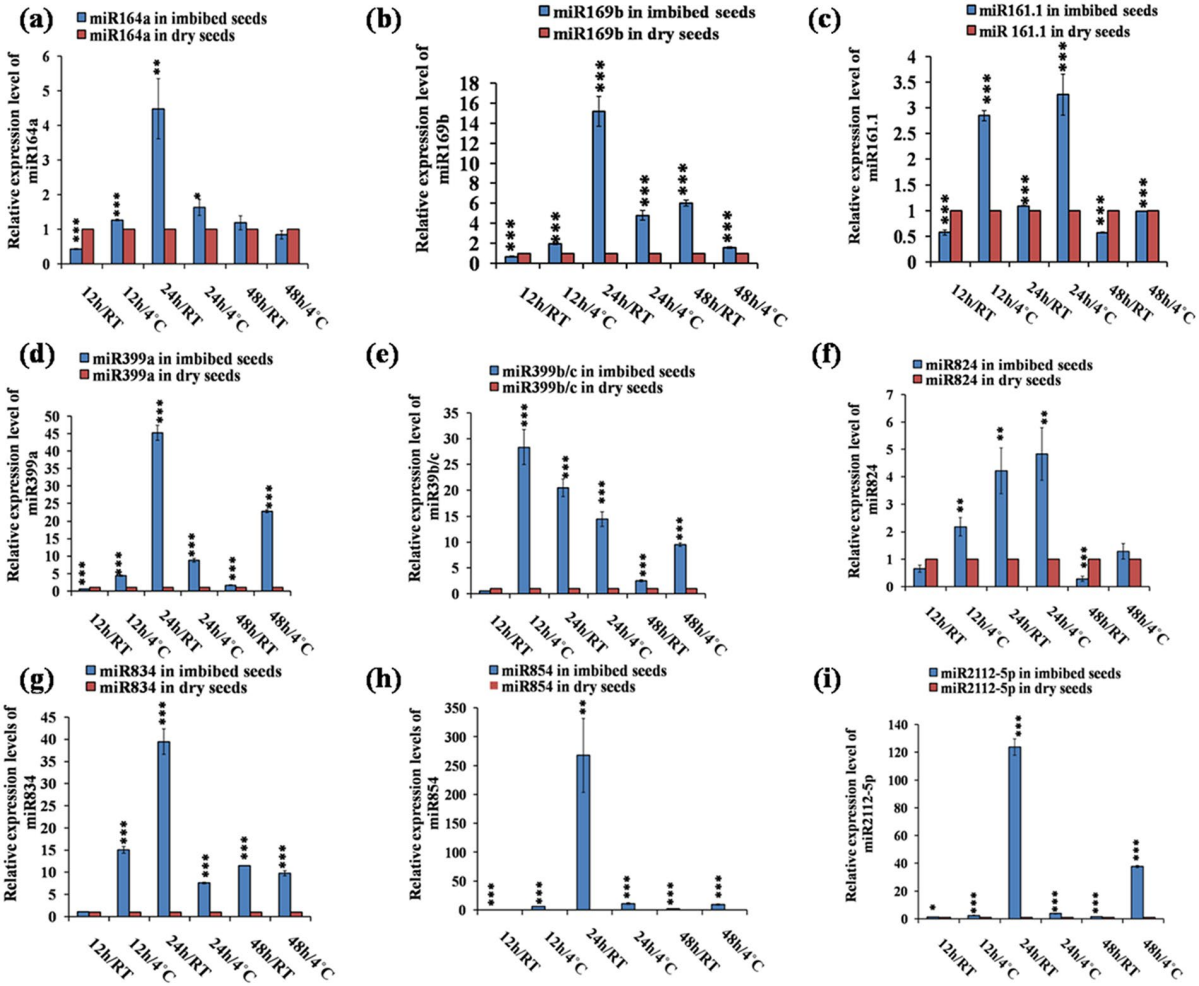
Quantitative RT-PCR based validation of expression of mature miRNAs in imbibed seeds, in comparison to dry seeds, in *Arabidopsis thaliana*. The qRT-PCR validation of miRNAs were done at six different germination conditions as 12 h/RT, 12 h/4 °C, 24 h/RT, 24 h/4 °C, 48 h/RT and 48 h/4 °C each compared to dry seed. The expression values presented here were the means of three biological replicates ± SD. The *Arabidopsis ACTIN7* was used for each sample as an endogenous control. (**a**) ath-miR164a; (**b**) ath-miR169b; (**c**) ath-miR161.1; (**d**) ath-miR399a; (**e**) ath-miR399b/or, c (in *Arabidopsis*, miR399b and c contain the same mature sequences); (**f**) ath-miR824; (**g**) ath-miR834; (**h**) ath-miR854; (**i**) ath-miR2112-5p.Asterisks indicate significant statistical differences: ***P < 0.001, **P < 0.01, *P < 0.05 (One-way ANOVA).

Through Venn diagram representation (Fig. 1d), we observed that 10 miRNA genes were common in both IS-4 °C vs DS and IS-RT vs. DS and they are miR161.2; miR169b, d, e; miR399a, b; miR824 and miR854a, c, e. Similarly, 8 miRNA genes were common in IS-4 °C vs. DS and IS-4 °C vs. RT and they are miR160b, c; miR161.2; miR164a, miR395a; miR447a and miR854a; while 6 miRNA genes were common in IS-RT vs. DS and 4 °C vs. RT and they are miR157c; miR161.2; miR834 and miR854a, b, d. Only two miRNAs, miR161.2 and miR854a, were common in all three cases here. Analysis of the data showed that amongst the differently expressed miRNAs, most abundant families were miR169 and miR854, with 11 and 5 family members, respectively (Fig. 1e). Among these miRNAs, eighteen precursor miRNA families were selected based on their p-values (≤0.05) and fold change values (≥2.0) across the three data sets for validation. Among these eighteen precursor miRNA, miR157a, miR157c, miR157d consist of same mature sequence; so we took only one mature sequence of miR157 (Fig. 2f) among the three. Also miR399b and miR399c contain the same mature sequence in *Arabidopsis*, therefore we had chosen only one among them (Fig. 3e). The other miRNAs for validation were miR165/166 (Fig. 2a), miR172a (Fig. 2b), miR390b (Fig. 2c), miR160a (Fig. 2d), miR156h (Fig. 2e), miR164a (Fig. 3a), miR169b (Fig. 3b), miR161.1 (Fig. 3c), miR399a (Fig. 3d), miR824 (Fig. 3f), miR834 (Fig. 3g), miR854 (Fig. 3h) and miR2112-5p (Fig. 3i).

### Validation of expression reflects dynamic regulation of miRNAs during seed germination

The different time points used for validation of miRNAs were 0 h (DS), 12 h/RT, 12 h/4 °C, 24 h/RT, 24 h/4 °C, 48 h/RT and 48 h/4 °C. The highest expression of miR165/166 was observed in case of 12 h/RT (Fig. 2a), which drastically changed in case of 12 h/4 °C. The lowest expressions were observed in 24 h/RT and 48 h/RT (Fig. 2a). However, it showed high expression at 24 h/4 °C and nearly equal expression to DS at 48 h/4 °C (Fig. 2a). We observed highest expression level of miR172a (Fig. 2b) during 24 h/4 °C and then 12 h/4 °C compare to DS (Fig. 2b), and the level reduced at the later stage of germination at 48 h/4 °C (Fig. 2b). Interestingly, its expression reduced at RT. We observed high level of expression of miR390 (Fig. 2c) compared to DS in almost all the conditions that we used. We found higher expression of miR390 (Fig. 2c) in both 24 h/RT and 24 h/4 °C conditions, and this expression level decreases at later stages of germination like 48 h (RT/4 °C) (Fig. 2c). The highest expression of miR160a was observed at 24 h/4 °C (Fig. 2d), then gradually in decreasing order at 24 h/RT, 12 h/4 °C, 12 h/RT compared to DS. We observed the lowest and almost same expression in case of 48 h/RT and 48 h/4 °C (Fig. 2d). Similar expression patterns were observed in case of both miR156h (Fig. 2e) and miR157a/c or, d (Fig. 2f). The miR157 families a, c and d constitute the same mature miRNA sequences. In both of the cases, we observed highest level of expression at 24 h/4 °C (2 fold in miR156 and ~5 fold in miR157). During cold imbibition, these miRNAs showed higher expressions compared to RT (Fig. 2e,f). Both the miRNAs showed reduced expression pattern with increasing time. We found maximum expression of miR164a (~4.5 fold) at 24 h/RT (Fig. 3a) and lowest expression at 12 h/RT (Fig. 3a). Also during 24 h cold imbibed condition, miR164a was showing upregulation compare to DS (Fig. 3a). Mainly, during very early cold imbibition stages (12 h, 24 h) miR164a showed high expression (Fig. 3a) but it gradually decreases at the later stages (48 h). Except 12 h/RT, in other germination conditions we observed upregulation of miR169b (Fig. 3b). The maximum expression of this miRNA was observed at 24 h/RT (Fig. 3b) like miR164a (Fig. 3a). The highest expression of miR161.1 was observed at 24 h/4 °C and then 12 h/4 °C (Fig. 3c), and the lower expressions were observed at 48 h/RT and 12 h/RT, which were almost same (Fig. 3c). At 48 h/4 °C, almost similar expression of miR169b was observed in both dry and imbibed seeds. Interestingly, in spite of single nucleotide difference in miR399a and miR399b/c, we observed a significant variation in their expression levels at different germination stages. Highest expression of miR399a (Fig. 3d) was at 24 h/RT and then 48 h/4 °C, whereas highest expression of miR399b/c (Fig. 3e) was at 12 h/4 °C and then 24 h/RT. But expression of both of the miRNAs was less at 12 h/RT and 48 h/RT compared to DS. Interestingly, all the miR399 (a, b and c) were induced in case of cold imbibition (4 °C) rather than RT. This indicates that cold imbibition plays significant role in miR399 expression during seed germination. In this study, we found higher expression of miR824 (Fig. 3f) in almost every stages, especially in cold imbibed conditions. We observed the highest expression of this miRNA at 24 h/4 °C (Fig. 3f). Although, miR834 was highly up regulated in every stages of germination compared to DS (Fig. 3g) and the maximum expression level (~40 fold) was observed at 24 h/RT (Fig. 3g). In case of miR854 (Fig. 3h) and miR2112-5p (Fig. 3i), highest expression of ~275 fold and ~130 fold respectively were observed at 24 h/RT. Overall, the expression pattern of most mature miRNAs were dynamically and differentially regulated at different time points under various germination conditions. Since miRNAs negatively regulate their targets, these results indicated possible differential regulation of their targets as well, which we further tested.

### Differential expression of miRNAs negatively correlates the expression of target transcripts during seed germination conditions

The targets of the validated miRNAs were identified through “psRNATarget” tool^25^. Our prediction indicated more than one targets for each miRNA under standard settings of prediction tool. Some of the targets were novel and not indicated earlier. For experimental validation of miRNA-target expression correlation, we chose the targets for each miRNA considering its respective expectation value of 0.5 to 2.5 range. In cases where more than two significant targets were identified, all of them were used in the validation experiments. Target gene specific primers were designed in the region flanking to miRNA binding site. The list of selected targets and target gene specific primers used in this study were provided in (supplementary table S2 and S3).We analysed the expression of total 27 targets of 15 differentially expressed (dE) miRNAs using fast SYBR green fluorescent based qRT-PCR.

**Figure 4:**
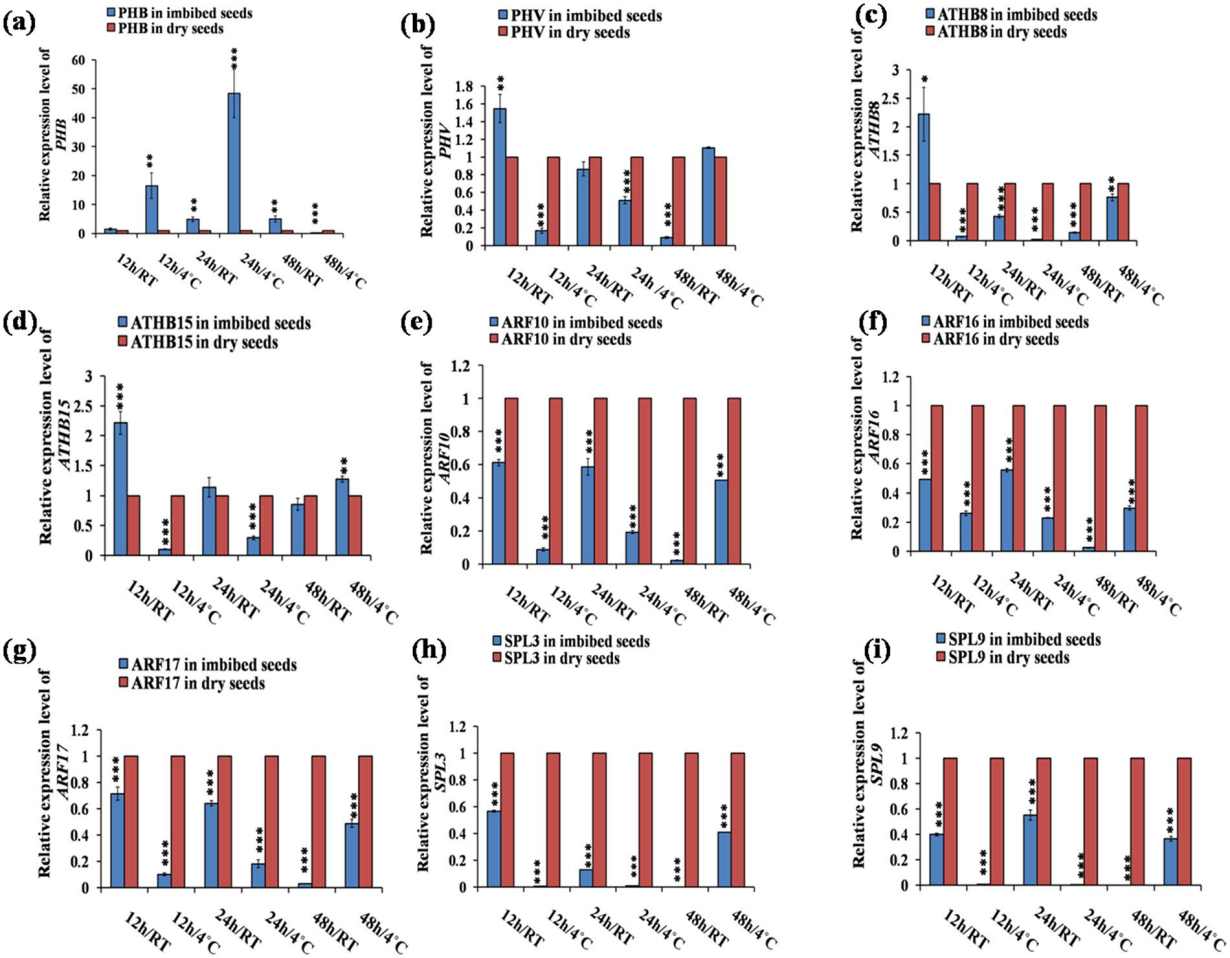
Quantitative RT-PCR based validation of different target genes in imbibed seeds in comparison to dry seeds in *Arabidopsis thaliana*. The qRT-PCR validation of miRNAs and the targets were done at six different germination stages as 12 h/RT, 12 h/4 °C, 24 h/RT, 24 h/4 °C, 48 h/RT and 48 h/4 °C each compared to dry seed. The expression values presented here were the means of three biological replicates ± SD. The *Arabidopsis ACTIN7* was used for each sample as an endogenous control.(**a–d**) targets of miR165/166; (**a**) *PHB*; (**b**) *PHV*; (**c**) *ATHB8*; (**d**) *ATHB15*. (**e–g**) targets of miR160; (**e**) *ARF10*; (**f**) *ARF16*; (**g**) *ARF17*. (**h,i**) targets of miR156/or, miR157; (**h**) *SPL3; (***i***) SPL9*. Asterisks indicate significant statistical differences, ***P < 0.001, **P < 0.01, *P < 0.05 (One-way ANOVA).

**Figure 5:**
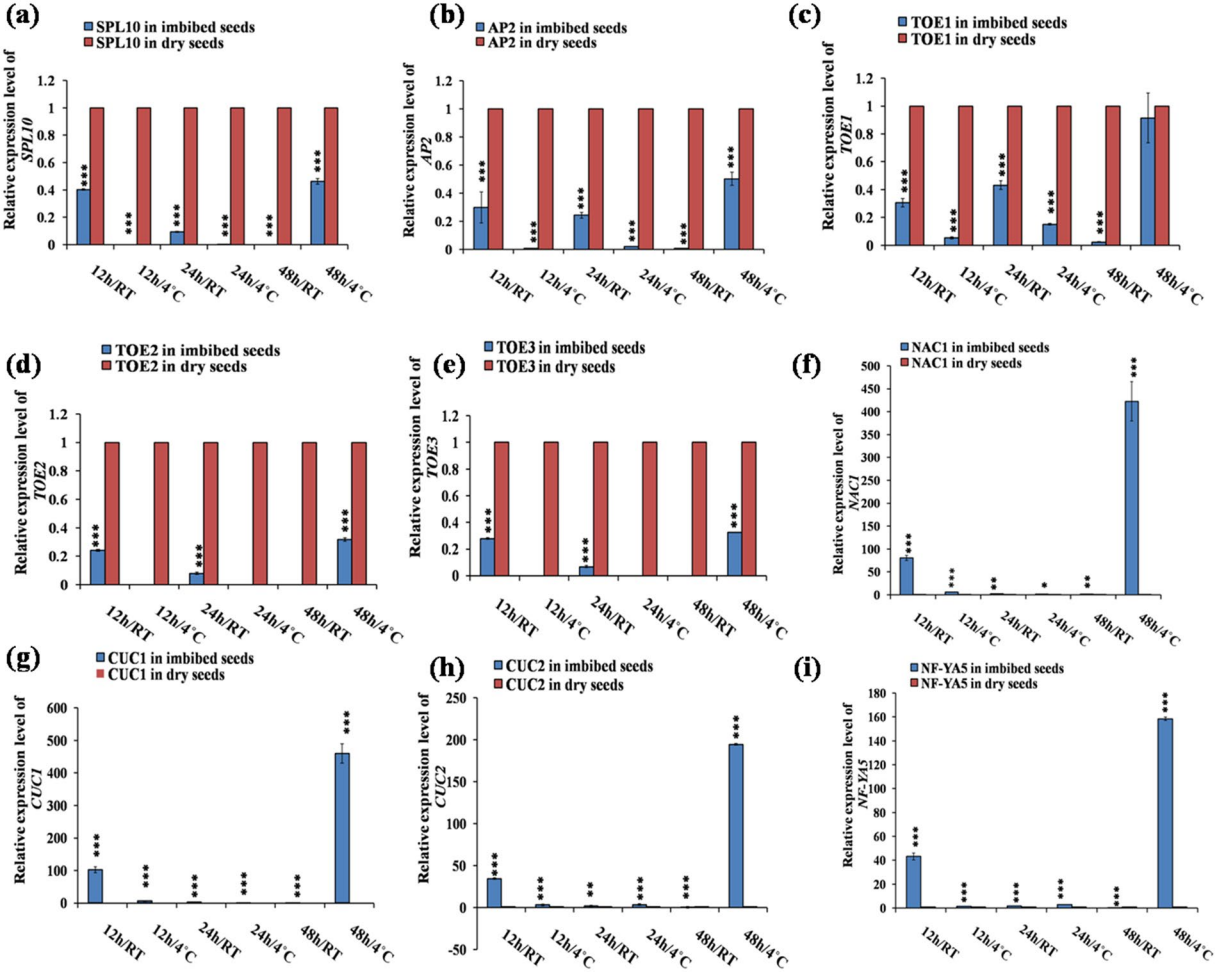
Quantitative RT-PCR based validation of various predicted target genes in imbibed vs dry seeds in *Arabidopsis*. The target genes were selected through psRNATarget tool based on their expectation values. The qRT-PCR based validation of the targets were done at six different germination stages, like 12 h/RT, 12 h/4 °C, 24 h/RT, 24 h/4 °C, 48 h/RT and 48 h/4 °C, each compared to dry seed. The expression values presented here were the means of three biological replicates ± SD. The *Arabidopsis ACTIN7* was used as an endogenous control for each sample. **(a)** *SPL10*- target of miR156/or, miR157. **(b–e)** targets of miR172; **(b)** *AP2*; **(c)** *TOE1*; **(d)** *TOE2*; **(e)** *TOE3*. **(f–h)** targets of miR164; **(f)** *NAC1*; **(g)** *CUC1*; **(h)** *CUC2*; **(i)** *NF-YA5*- target of miR169. Asterisks indicate significant statistical differences; ***P < 0.001, **P < 0.01, *P < 0.05 (One-way ANOVA).

**Figure 6:**
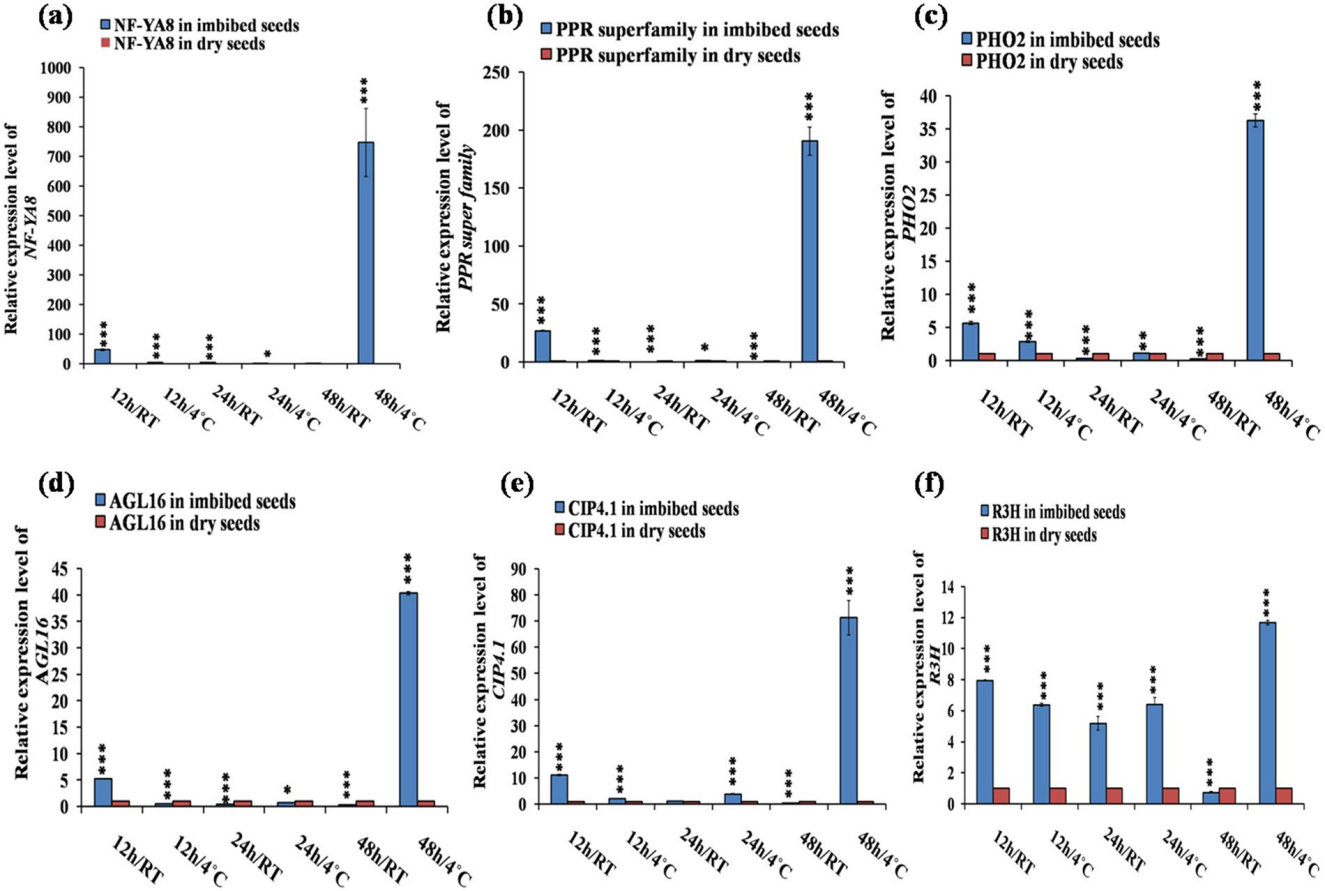
Quantitative RT-PCR based validation of various predicted target genes in imbibed vs dry seeds in *Arabidopsis*. The target genes were selected through psRNA Target tool based on their expectation values. The qRT-PCR validation of the targets were done at six different germination stages as 12 h/RT, 12 h/4 °C, 24 h/RT, 24 h/4 °C, 48 h/RT and 48 h/4 °C each compared to dry seed. The expression values presented here were the means of three biological replicates ± SD. The *Arabidopsis ACTIN7* was used for each sample as an endogenous control. Expression of **(a)** *NF-YA8****-***target of miR169; **(b)** *PPR superfamily*- target of miR161.1; **(c)** *PHO2*- target of miR399; **(d)** *AGL16*- target of miR824; **(e)** *CIP4.1 or CIP4*- target of miR834; **(f)** *R3H*- target of miR854. Asterisks indicate significant statistical differences; ***P < 0.001, **P < 0.01, *P < 0.05 (One-way ANOVA).

The expression of the targets was determined using the same germination conditions as those for the miRNAs, and in most of the cases we obtained opposite expression of the targets compare to their corresponding miRNAs. This inverse correlation partially validated the miRNA targets, since miRNAs mostly post-transcriptionally cleave their respective target gene transcripts. However, the inverse relationship of expression pattern between miRNAs and their targets were limited in some cases during specific stages of germination, which might be because of condition induced transcriptional regulation. We validated the expression of *PHB (PHABULOSA)* (Fig. 4a), *PHV (PHAVOLUTA)* (Fig. 4b), *ATHB8 (Arabidopsis thaliana HOMEOBOX GENE8)* (Fig. 4c) and *ATHB15 (Arabidopsis thaliana HOMEOBOX GENE15)* (Fig. 4d), which are targets of miR165/166. In cold imbibition (4 °C) condition at 12 h, miR165 expression was less compared to DS, where as *PHB* (Fig. 4a) expression was high. At 48 h/4 °C, miR165 expression was upregulated compared to DS but the expression of *PHB* was severely decreased in 48 h/4 °C (Fig. 4a). We found maximum expression of *PHB* in 24 h/4 °C. The expression of *PHB* was slightly different as compare to *PHV*, *ATHB8* and *ATHB15*, since both *PHV, ATHB8* and *ATHB15* showed high expressions at 12 h/RT whereas *PHV* expression was reduced (Fig. 4a–d). The expressions of *ARF10* (Fig. 4e), *ARF 16* (Fig. 4f) and *ARF 17* (Fig. 4g) as the target of miR160 were highly downregulated in all germination stages compare to DS. We analysed the expression of *SPL*3 (*SQUAMOSA PROMOTER BINDING PROTEIN LIKE3*) (Fig. 4h), *SPL*9 (Fig. 4i) and *SPL*10 (Fig. 5a), which are the targets of both miR156/157. We observed low transcript level of the target genes *SPL*3, 9 and 10 in all the germination conditions as compared to DS. However, the transcript level started to increase in the later stage of germination at 48 h/4 °C. We validated the expression of 4 targets of miR172, namely *AP2 (APETALA2), TOE1 (TARGET OF EARLY ACTIVATION TAGGED*1)*, TOE2, TOE3* out of 6. We observed highest expression of the targets at 48 h/4 °C (Fig. 5b–e). Comparatively, high expression of these targets was observed in case of RT, rather than cold imbibition (4 °C), which inversely correlates with increased expression of miR172 under cold imbibitions. We validated expression of three different targets of miR164, such as *NAC1 (NO APICAL MERISTEM1)* (Fig. 5f), *CUC1 (CUP SHAPED COTYLEDON1)* (Fig. 5g) and *CUC2* (Fig. 5h). The maximum transcript level of these targets was observed in 48 h/4 °C and followed by expression in 12 h/RT. In these conditions, the expression level of *NAC1* was ~430 fold, whereas *CUC1* and *CUC2* expression was induced by ~450 and ~190 folds respectively. In other conditions, we found low expression of these targets. For miR169, we validated the expression of two targets - *NF-YA5 (NUCLEAR FACTOR Y, SUBUNIT A5)* and *NF-YA8*. Like the targets of miR164a, we mostly observed upregulation of expression of the targets of miR169b in 48 h/4 °C and 12 h/RT (Figs 5i, 6a), and highest transcript level was observed in 48 h/4 °C (Figs 5i, 6a). The significantly high transcript level at 48 h/4 °C in case of *NF-YA5* was 160 fold, and for *NF-YA8* it was ~750 fold. We validated expression of miR161 target *PPR (PENTATRICOPEPTIDE REPEAT)* super family (Fig. 6b). The highest expression of the target was observed at 48 h/4 °C (Fig. 6b), and the transcript level was observed as ~190 fold. The second upregulation of this target was observed at 12 h/RT (~30 fold). In other cases were observed either same or downregulation of the targets as compared to DS. *PHO2 (PHOSPHATE 2)* expression correlation was validated as the target of miR399 (Fig. 6c). The highest transcript level of the target was observed at 48 h/4 °C (~37 fold). The second upregulation was observed at 12 h/RT (~6 fold) and then 12 h/4 °C (~2.5 fold). Expression in other conditions showed either downregulation or almost similar transcript level w.r.t. DS (Fig. 6c). We validated the expression correlation *AGL16 (AGAMOUS-like 16)* (Fig. 6d), *CIP4.1(COP1-interacting protein4.1)* (Fig. 6e) and *R3H* (Fig. 6f) as the targets of miR824, miR834 and miR854 respectively. The maximum target transcript level of *AGL16* was observed at 48 h/4 °C (~41 fold) and then 12 h/RT (~5 fold). In other cases, the downregulation of the targets were observed. The highest transcript level for *CIP4.1* and *R3H* were observed at 48 h/4 °C (Fig. 6e,f). It was ~70 fold for *CIP4.1* and ~12 fold for *R3H*. We observed upregulation of the target w.r.t. DS in almost all conditions except 48 h/RT.

### Expression pattern of miR390b in seeds correlates with the expression of tasiR-*ARF* target *ARF2/3/4*, and indicates its role in seed germination

**Figure 7:**
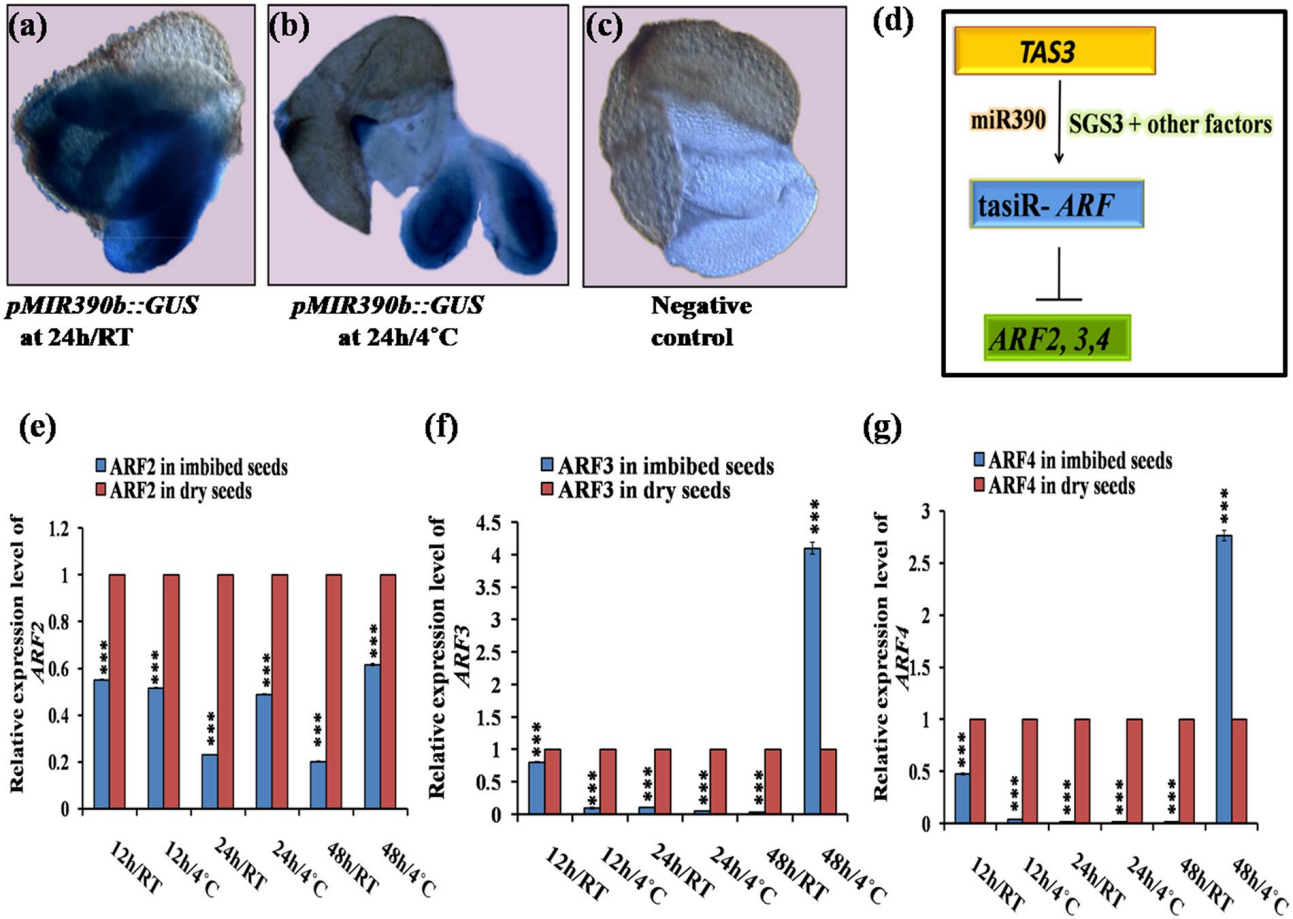
Spatial expression of *pMIR390b::GUS* in germinating seeds, function of miR390, and expression of *ARF2, ARF3*, and *ARF4* during seed germination. (**a**) *GUS* expression of *pMIR390b::GUS* at 24 h/RT imbibed condition; (**b**) *GUS* expression of *pMIR390b::GUS* at 24 h/4 °C imbibed condition; (**c**) Negative control of *GUS* assay at 24 h/4 °C imbibed Col-0 seed. 24 h/RT-imbibed seeds were showing the higher expression of miR390b compare to 24 h/4 °C-imbibed seeds, which is similar to the expression pattern of the qRT-PCR based validation result of miR390b; (**d**)The model depicting the role of miR390 inthe biogenesis of tasiR-*ARFs* and regulation of *ARF2/3/4* (According to Marin *et al*., 2010). (**e–g**) qRT-PCR based expression pattern of *ARF2/3/4*, which are the targets of tasiR-*ARF;* (**e**) transcript level of target *ARF2*; (**f**) transcript level of target*ARF3;* (**g**) transcript level of target *ARF4*.

Since miR390 is required for *TAS3* transcript and ta-siRNA production, we chose to chracterise it further to understand possible involvement of miRNA-*tasiR-ARF* module in seed germination. We have found maximum expression of miR390b at 24 h/RT following 24 h/4 °C through qRT PCR assay (Fig. 2c). To verify the expression at tissue level, we performed histochemical *GUS* assay with seeds of *pMIR390b::GUS* homozygous line along with the WT (Col-0) seeds negative control. We observed high level of expressions of miR390b at 24 h/RT (Fig. 7a) and then 24 h/4 °C (Fig. 7b) of germinating seeds, where as Col-0 as a negative control, showing no expressions (Fig. 7c). Since miR390 is essential for maturation of *TAS3* and regulate the production of functional tasiR-*ARF*, its expression should regulate transcript level of tasiR-*ARF* targets *ARF2, ARF3* and *ARF4* through different feedback mechanisms^24^. We observed that the transcript levels of targets *ARF2/3/4* were low at the early stages of germination and became high in later stage at 48 h/4 °C (Fig. 7e, f and g). However, interestingly, at 48 h/RT, the expression of the targets *ARF2/3/4* was not high. Expression of *ARF3* (Fig. 7f) and *ARF4* (Fig. 7g) transcript level increased significantly at 48 h/4 °C (for *ARF3* it was 4 fold, and for *ARF4* it was ~2.75 fold).

In the previous study, it was reported that first 24 h (Phase-II of triphasic seed germination events) of imbibition is very critical for seed germination, because of maximum cellular repairments, RNA transcription and metabolism resumptions occur in this phase^26–28^. Our results indicate potential role of miR390-tasiR-*ARF* module in seed germination process.

## Discussion

In higher plants, seed germination is one of the most vital phase transition from seed to seedling stage^2^. Although, small RNAs have been implicated in various aspects of plant development, the regulatory role of small RNAs in seed germination is less explored area till date. However, few recent reports indicated involvement of miRNAs in different plant species such as rice, maize and other monocot but in *Arabidopsis*, whether and how small RNAs might be regulating dynamic process of seed germination is largely unknown to us. Our present study focused on the identification and characterization of miRNAs mediated gene regulation in early stages of seed germination in *Arabidopsis thaliana*.

### miRNAs and their targets are dynamically regulated at different conditions during germination

We have identified 58 miRNA precursors (pre-miRNAs) belonging to 30 miRNA families to be differently expressed in comparative study of three different conditions – (1) IS-4 °C vs. DS (Fig. 1a), (2) IS-RT vs. DS (Fig. 1b) and (3) IS-4 °C vs. IS-RT (Fig. 1c) during germination. Among these, 15 miRNA precursors belonging to 14 families were of P ≤ 0.05 and fold change ≥ 2.0 and considered to be significant. Since mature miRNAs are the main functional molecule that regulates their targets, we observed their dynamic regulation using SL-qRT-PCR at three different time points (12 h, 24 h and 48 h) of germination conditions. Expression of miR399a and miR399b/c were analyzed independently, since their mature sequence differed.

We observed significant upregulaion of miR165/166 in 12 h/RT and downregulation at 24 h/RT and 48 h/RT (Fig. 2a). The expression of target *PHB* was downregulated at 12 h/RT (Fig. 4a), where as it was upregulated at 24 h/RT and 48 h/RT (Fig. 4a), indicating their post-transcriptional regulation by miR165/166 in these conditions. Additionally, *ATHB15* was also slightly upregulated in 24 h/RT (Fig. 4d). Previous reports implicated miR165/166 module in leaf, shoot, root vascular patterning (Zhou *et al*., 2007) and also in seed germination in maize^20^ and rice^21^. Previous reports indicated role of miR172 in regulation of vegetative to reproductive phase change and cold stress induced response affecting root growth^29,30^. We observed that miR172a was significantly upregulated in 24 h/4 °C (Fig. 2b) and downregulated in 24 h/RT and 48 h/4 °C (Fig. 2b), whereas its target *AP2* (Fig. 5b)*, TOE1* (Fig. 5c*), TOE2* (Fig. 5d*)* and *TOE3* (Fig. 5e*)* were significantly downregulated at 24 h/4 °C. This inverse correlation of expression of miR172a and its targets indicate their post-transcriptional regulation by miRNAs. Our results indicate possible role of miR172-target *AP2*/*TOE*s module in seed germination process. miR160 is involved in auxin signalling pathway during various plant growth and developmental processes^14,15,31^ by negatively regulating its target transcription factors *ARF10*, *ARF16* and *ARF17* via hormonal crosstalk^32^. We observed the significant upregulation of miR160 during all of the germination conditions we used and found maximum upregulation at 24 h/4 °C followed by 24 h/RT (Fig. 2d). This consistent upregulation of miR160 led to the consistent downregulation of its targets *ARF10*/*16*/*17* (Fig. 4e, f, g) during all germination conditions. This indicates the potential significant role of miR160 during early stages of seed germination irrespective of their stratification status. The downregulation of *ARFs* by miR160 during imbibition indicates possible auxin-ABA crosstalk during germination since ABA became downregulated in over expressing miR160 plants^15^ and *ARFs* are known to be involved in auxin signalling. Earlier findings also indicate the role of miR160 in seed germination in rice^21^ and *Nelumbo nucifera*^22^. According to the previous report, miR156 and its closely related miR157^33^ are the principal regulators of transition from juvenile to adult phase. They are also involved in shoot development, floral induction, initiation of leaf etc^34^ by negatively regulating their target *SPLs*. Among the ten *SPL*s in *Arabidopsis*, we chose *SPL3* (Fig. 4h), *SPL9* (Fig. 4i) and *SPL10* (Fig. 5a), since they are the targets of both miR156 and miR157. We observed significant upregulation of these two miRNAs at 24 h/4 °C, followed by 12 h/4 °C (Fig. 2e,f); and the significant downregulation of their targets in the same above said germination conditions. This inverse correlation of miR156/157 and its target *SPLs* indicate their post-transcriptional regulation. Earlier studies also indicated the role of miR156 in the dynamic seed germination process of maize^20^ and *Nelumbo nucifera*^22^. miR164 plays a significant role in formation of proper organ boundaries^35^, floral patterning^36^, leaf morphogenesis^37^ and lateral root development^38^ by negative regulation of its target *NAC1*, *CUC1* and *CUC2*. We observed the maximum significant upregulation of miR164a at 24 h/RT (Fig. 3a), followed by 24 h/4 °C (Fig. 3a), which was further followed by 12 h/4 °C (Fig. 3a). Here we also observed the inverse correlation of miRNA–target. We found maximum significant upregulation of the targets at 48 h/4 °C, followed by 12 h/RT (Fig. 5f–h). The inverse correlation of target- miRNA indicates the post-transcriptional regulation by miRNAs. In the previous report, there is the indication of the involvement of miR164 in seed germination in maize^20^. The miR169 family is the largest miRNA family in *Arabidopsis* and is encoded by 14 members^39^; however, only a few members have been annotated with specific functions. The miR169 targets members of the *Arabidopsis NF-YA* gene family^40^. *NF-Y* encodes a CCAAT-binding transcription factor, which participates in transcriptional regulation of a large number of genes^41^. In *Arabidopsis*, there are 10 genes coding for the *AtNF-YA* subunit^42^. It was reported that over expression of *NF-YA5* caused hypersensitivity to ABA during seed germination^43,44^. We observed the maximum significant upregulation of miR169b at 24 h/RT (Fig. 3b), followed by 48 h/RT (Fig. 3b); and we observed maximum significant downregulation in the above said germination conditions and maximum upregulation of the targets *NF-YA5* and *NF-YA8* at 48 h/4 °C, followed by 12 h/RT (Figs 5i, 6a). This inverse correlation of target-miRNA indicates the post-transcriptional regulation of miRNA. The miR161 is a non conserved miRNA, since it is represented by single genes rather than multigene families^45^. The miR161 locus is unusual as it encodes overlapping miRNAs (miR161.1 and miR161.2) from a single precursor sequence^45^. miR161.1 targets *PPR* superfamily through negative regulation, which has a major impact on evolutionary background^46^. We observed the maximum significant expression of miR161.1 during 24 h/4 °C (Fig. 3c), followed by 12 h/4 °C (Fig. 3c); and we observed the maximum significant downregulation of the target *PPR* superfamily at 24 h/4 °C (Fig. 6b), then 12 h/4 °C (Fig. 6b). This inverse correlation of miR161.1 and its target PPR superfamily indicates the post- transcriptional regulation of miRNA. Our result indicates the role of miR161.1 –target *PPR* superfamily module in seed germination process. The *Arabidopsis* genome encodes six miR399 genes (miR399a -f), which are all induced by Phosphorus starvation to different extents^47^. miR399 is involved in orthophosphate (Pi) deficiency signalling pathway targets *PHOSPHATE2 (PHO2)* gene encoding E2 enzyme that negatively regulates phosphate uptake and root-to-shoot allocation^47,48^. The miR399a and miR399b/c (miR399b and miR399c have same mature sequence in *Arabidopsis*) has single nucleotide difference in the 13^th^ position from 5’ end. Throughout the germination stages miR399a and miR399b/c expression levels were upregulated in comparison to DS and the target *PHO2* expression level was downregulated, except 48 h/4 °C (Fig. 6c). Previous reports also indicated the expression of miR399 in seeds of maize^20^ and *Nelumbo nucifera*^22^. Availability of phytate and orthophosphate (Pi) in germinating seeds may regulate miR399 expression, which is known to be regulated by Pi availability, during germination^47^. miR824 is *Brassicaceae*-specific miRNA^9,49,50^. miR824 has function in rosette and cauline leaves, shoots, inflorescence and roots^50,51^ by negative regulation of its target *AGAMOUS-LIKE16 (AGL16)*, which encodes a MADS box transcription factor^49,52^. We observed the maximum significant upregulation of miR824 during 24 h/4 °C (Fig. 3f), followed by 24 h/RT (Fig. 3f), which is further followed by 12 h/4 °C (Fig. 3f). We observed the significant downregulation of the target also. At 48 h/4 °C, followed by 12 h/RT (Fig. 6d), we observed the significant upregulation of the target. The inverse correlation of miR824 and its target indicates their post-transcriptional regulation by miRNA. Our results indicate the role of miR824-target *AGL16* module in seed germination process. miR834 is a non-conserved miRNA. The predicted targets of miR834 are *DEMETER-LIKE 2 (DML2)* and *COP1-INTERACTING PROTEIN1 (CIP1)*. In this study we have validated *CIP4.1(AT4G00930.1)*. CIP1was the first reported interacting protein for *CONSTITUTIVE PHOTOMORPHOGENIC 1 (COP1)* of *Arabidopsis*^53^. *CIP1* is a positive regulator of abscissic acid (ABA) response^53^. CIP4, a homologue of CIP4.1, is CIP4 is COP1 interactive partner and acts downstream of *COP1*^54^. We observed the significant upregulation of miR834 in all of the germination conditions that we used irrespective of stratification status; and found maximum significant upregulation during 24 h/RT (Fig. 3g), followed by 12 h/4 °C (Fig. 3g). We found maximum significant downregulation of target *CIP4.1* during 48 h/RT, followed by 12 h/4 °C (Fig. 6e). The inverse correlation of miRNA-target also indicates the post-transcriptional regulation by miRNA and the miR834-target *CIP4.1* module in the dynamic seed germination process. miR854 is a conserved and stress responsive^55^ miRNA. Plants and animals share miRNAs of the miR854 family, suggesting a common origin of these miRNAs as regulators of basal transcriptional mechanisms^56^. Recently, it was shown that miR854 regulated the rhizome development and the essential oil biosynthesis in ginger^57^. We observed significant downregulation of miR854 expression in most of the germination conditions, except 24 h/RT (Fig. 3h), we observed the inverse correlation of miRNA and its target *R3H* (Fig. 6f), which indicates the post-transcriptional regulation by miRNA and miR854-*R3H* module in seed germination. miR2112- 5p is a non-conserved miRNA. We observed the maximum significant expression of this miRNA during 24 h/RT (Fig. 3i), followed by 48 h/4 °C (Fig. 3i). According to psRNATarget tool, we found two targets of miR2112-5p. One is ergosterol biosynthesis ERG4/ERG24 family and another is pentatricopeptide repeat PPR superfamily protein. miR2112- 5p targets both of the target proteins through translational inhibition.

We observed upregulation of most of the miRNAs in 24 h imbibition, which indicates their role in seed germination during imbibition onwards in *Arabidopsis thaliana*. Some of the miRNAs showed the minor discrepancies between microarray data and the qRT-PCR results. The reason behind that is, during microarray we had pulled the RNAs of both RT and 4 °C imbibed seeds individually. Another reason could be the differences in the specificity, sensitivity and algorithm used between the two techniques. In most of the cases we found higher accumulation of the targets during 48 h and 12 h of germination conditions. In some conditions we didn’t observe the inverse correlation between miRNAs and their targets, this indicates seed germination condition induced transcriptional regulation of these targets, besides their post-transcriptional regulation by miRNAs. This further implies that both transcriptional and post-transcriptional regulation of miRNA targets play important role in seed germination process.

### Expression pattern of miR390 and downstream *ARF2/3/4* indicates potential role of miRNA-ta-siRNA crosstalk in seed germination process

Spatial expression pattern of *pMIR390b::GUS* in embryonic root, cotyledon and endosperm of germinating seeds (Fig. 7a,b) indicates its potential role in seed germination. We observed that the induced expression of miR390b (Fig. 2c) correlates downregulation of *tasiR-ARF* targets *ARF2, ARF3* and *ARF4* in early stages of seed germination (Fig. 7e,f and g). Upregulation of miR390 should enhance the production of *tasiR-ARF* leading to the down regulation of its target *ARF2/3/4*, which we observed in most of the germination conditions we used. This result indicates the role of miR390-tasiR-*ARF* mediated post-transcriptional regulation and a crosstalk of two classes of small RNAs (miRNA and ta-siRNA) in seed germination. Although we cannot rule out additional involvement of other ta-siRNAs. Thus, our study indicates that the miR390 – tasiR-*ARF - ARF2/3/4* module and crosstalk of miRNA and ta-siRNA pathways (Fig. 7d) contributes to the regulation of the dynamic process of seed germination in *Arabidopsis*, besides role of other miRNAs and their targets.

## Methods

### Plant material and growth conditions

*Arabidopsis thaliana* ecotype Col-0 was used throughout the study. Seeds were surface sterilized and germinated on half-strength Murashige and Skoog (MS) medium (HiMedia) supplemented with 1% sucrose and 0.8% agar^58^. The plates were kept at 4 °C in dark for 3 days for stratification and then transferred to normal growth conditions. Plants were grown in a controlled environment at 22 ± 2 °C, under 16:8 h light (~120 µmol m^−2^ s^−1^)/dark photoperiodic cycle. The *pMIR390b::GUS* was described earlier^23^; Col-0 seeds were obtained from ABRC.

### Total RNA extraction and cDNA synthesis

Total RNA was extracted from germinating wild type Col-0 *Arabidopsis* seeds at 0 (dry seeds), 12, 24 and 48 h after imbibition both at room temperature (RT) and 4 °C, using slightly modified guanidine hydrochloride method^59^. The integrity, quantity and quality of the RNAs were assessed by Bio Analyser 2100 (Agilent Technologies), Nanodrop spectrophotometer (Thermo Fisher Scientific) and also in 1.2% TAE agarose gel (Supplementary Fig. S1a) and in MOPS-formaldehyde gel (Supplementary Fig. S1b). The total RNA was further treated with DNase-I (Thermo Fisher Scientific, USA) and purified with lithium chloride (Sigma). First-strand cDNA synthesis was performed using 500ng of purified RNA with Super Script III reverse transcriptase (Invitrogen, USA) as per manufacturer’s instructions.

### miRNA microarray analysis

For miRNA microarray, total RNA was isolated, and the quality of RNA was tested as described above. Approximately 250 ng of total RNA was used from each sample in two biological replicates for setting up *in-vitro* transcription reaction (Affymetrix, USA). The RNA purification, fragmentation and hybridization reactions were done using *Arabidopsis* miRNA chip miRNA v1.0 array (Affymetrix, USA). Washing and scanning were performed as suggested in Affymetrix Gene Chip total RNA procedure. Results obtained after scanning were analyzed using Gene spring GX software v11.5. The processed raw signal intensities were subjected to normalization using the same software. Expression of differentially expressed and selected miRNA genes in microarray were further validated by real time stem-loop quantitative RT-PCR (qRT-PCR).

### **Stem-loop and regular quantitative RT- PCR (qRT- PCR)**

The expression level of mature miRNAs was analysed by stem-loop qRT-PCR, as described earlier^60–62^. SuperScript III reverse transcriptase (Invitrogen, USA) was used according to the manufacturer’s instructions and as described earlier^60^. The primer sequences used for stemloop and regular qRT-PCR were provided in (Supplementary Table S1 and S2). The transcript levels were normalized using *ACTIN7* (*ACT7*) as an endogenous control.

### **β-glucuronidase (GUS) histochemical assay**

During germination, the highest expression of miR390b was observed at 24 h germination condition after imbibition both at room temperature and 4 °C. To check this *in vitro*, we imbibed *pMIR390b::GUS* transgenic homozygous seeds 24 h in room temperature and 4 °C. After that, the germinating seeds were transferred to GUS staining buffer and staining was performed as described earlier^63^. The *GUS* expression was checked under stereomicroscope.

### Statistical analysis

For statistical analysis, we performed one way ANOVA to determine significant differences among the samples. Differences were taken as significant when P < 0.05.

## Electronic supplementary material

Supplementary Information
