# Supplementary material for: Expression dynamics of miRNAs and their targets in seed germination conditions reveals miRNA-ta-siRNA crosstalk as regulator of seed germination
Source: Sci Rep. 2018 Jan 19;8:1233. doi: 10.1038/s41598-017-18823-8 (PMC5775422; doi:10.1038/s41598-017-18823-8)
Supplement: Supplementary file 1 — Supplementary Information [file 41598_2017_18823_MOESM1_ESM.pdf]

Title of the article:

Expression dynamics of miRNAs and their targets in seed germination conditions reveals miRNA-ta-siRNA crosstalk as regulator of seed germination

Full name of Authors:

Shabari Sarkar Das[1], Sandeep Yadav[2], Archita Singh[2], Vibhav Gautam[2],Ananda K Sarkar[2] ,Asis K Nandi[3],Prakash Karmakar[3], Manoj Majee[2], and Neeti Sanan-Mishra[1]

Affiliation:

[1]International Centre for Genetic Engineering and Biotechnology,
Plant RNAi group,
Aruna Asaf Ali Marg,
New Delhi-110067, India

[2]National Institute of Plant Genome Research,
Aruna Asaf Ali marg,
New Delhi- 110067, India

[3]Department of Botany and Forestry,
Vidyasagar University, Midnapore,
West Bengal, India

Email addresses:

Shabari Sarkar Das (shabaridas@yahoo.co.in), Sandeep Yadav(sandeep_yadav@nipgr.ac.in), Archita Singh (archita@nipgr.ac.in), Vibhav Gautam (vibhav@nipgr.ac.in),Ananda K Sarkar (aksarkar@nipgr.ac.in),Asis K Nandi (aknind@mail.vidyasagar.ac.in),Prakash Karmakar (prakash@mail.vidyasagar.ac.in), Manoj Majee (manojmajee@nipgr.ac.in), Neeti Sanan Mishra (neeti@icgeb.res.in).

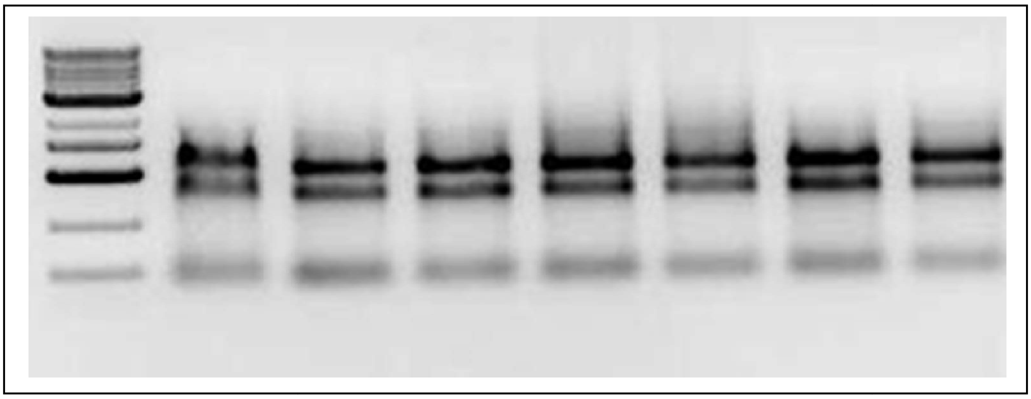

M 1 2 3 4 5 6 7

Fig. S1a. Total RNAs from *Arabidopsis* seeds in 1.2% TAE agarose gel.

(1: DS, 2: 12h/RT, 3: 12h/4˚C, 4: 24h/RT, 5: 24h/4˚C, 6: 48h/RT, 7: 48h/4˚C , (M): marker. Since this is a gel for RNA quality check, no marker is required.)

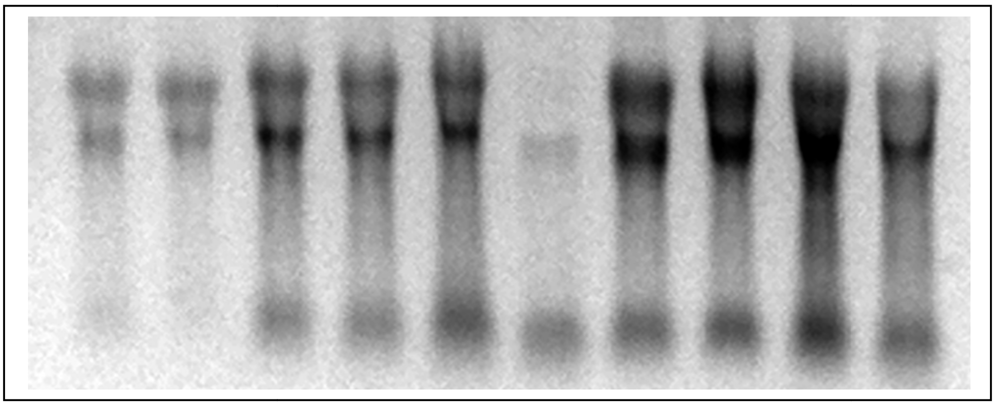

(a) (b) 1 2 3 (c) 4 5 6 7

Fig. S1b. Total RNAs from *Arabidopsis* seeds in MOPS-formaldehyde gel.

((a) and (b): Positive control RNAs from leaf and shoot tissues, (c): Empty lane having some RNA diffused into it from neighbouring well/s. 1: DS, 2: 12h/RT, 3: 12h/4˚C, 4: 24h/RT, 5: 24h/4˚C, 6: 48h/RT, 7: 48h/4˚C . Since this is a gel for RNA quality check, no marker is required.)

Supplementary Table S1. List of primers for stem loop qRT-PCR used in this study.

Serial no.	Name of the Primers	Sequence of the primers
1	ath-miR165/166-F	GTACTCGGACCAGGCTTCA
2	ath-miR165/166-SL	GTC GTA TCC AGT GCA GGG TCC GAG GTA TTC GCA CTG GAT ACG AC GGGGGA
3	ath-miR172(a/b)F	CGGCGAGAATCTTGATGATG
4	ath-miR172(a/b)-SL	GTC GTA TCC AGT GCA GGG TCC GAG GTA TTC GCA CTG GAT ACG AC ATGCAG
5	ath-miR390b-F	GCGTAAGCTCAGGAGGGAT
6	ath-miR390b-SL	GTC GTA TCC AGT GCA GGG TCC GAG GTA TTC GCA CTG GAT ACG ACGGCGCT
7	ath-miR160a	ACTGCCTGGCTCCCTGT
8	160a-SL	GTCGTATCCAGTGCAGGGTCCGAGGTATTCGCACTGGATACGACT GGCA
9	ath-miR156h-F	GCGTGGTGACAGAAGAAAGA
10	ath-miR156h-SL	GTC GTA TCC AGT GCA GGG TCC GAG GTA TTC GCA CTG GAT ACG AC GTGCTC
11	ath-miR157(a/c/d)-F	GCGTCGTTGACAGAAGATAGA
12	ath-miR157(a/c/d)-SL	GTC GTA TCC AGT GCA GGG TCC GAG GTA TTC GCA CTG GAT ACG AC GTGCTC
13	ath-miR164a	GCTAATGGAGAAGCAGGGCA
14	164a-SL	GTCGTATCCAGTGCAGGGTCCGAGGTATTCGCACTGGATACGACT GCAC
15	ath-miR169b	GCGTACAGCCAAGGATGACT
16	169b-SL	GTCGTATCCAGTGCAGGGTCCGAGGTATTCGCACTGGATACGACC CGGC
17	ath-miR161-F	GCTCGCTGAAAGTGACTACAT
18	ath-miR161-SL	GTC GTA TCC AGT GCA GGG TCC GAG GTA TTC GCA CTG GAT ACG AC ACCCCG
19	ath-miR399a-F	GCGATGCCAAAGGAGATTT
20	ath-miR399a-SL	GTC GTA TCC AGT GCA GGG TCC GAG GTA TTC GCA CTG GAT ACG AC CAGGGC
21	ath-miR399(b/c)F	ACGC TGCCAAAGGAGAGTT
22	ath-miR824-F	CTGCGCTAGACCATTTGTGAG
23	ath-miR824-SL	GTC GTA TCC AGT GCA GGG TCC GAG GTA TTC GCA CTG GAT ACG AC TCCCTT
24	ath-miR834-F	GTCGTGGTAGCAGTAGCGG
25	ath-miR834-SL	GTC GTA TCC AGT GCA GGG TCC GAG GTA TTC GCA CTG GAT ACG AC TTACCA
26	ath-miR854(a/b)-F	GCGTCAGATGAGGATAGGGAG

27	ath-miR854-SL	GTC GTA TCC AGT GCA GGG TCC GAG GTA TTC GCA CTG GAT ACG AC CTCCTC
28	ath-miR2112-5p-F	GCTTAGCGCAAATGCGGA
29	ath-miR2112-5p-SL	GTC GTA TCC AGT GCA GGG TCC GAG GTA TTC GCA CTG GAT ACG ACACATTG
30	Universal reverse primer (UVRP)	GTG CAG GGT CCG AGG T
31	ath-Actin7-F	GGT CGT ACA ACC GGT ATT GT
32	ath-Actin7-R	GAT AGC ATG TGG AAG TGA GAA

Supplementary Table S2. List of primers for validation of miRNA targets used in this study by qRT-PCR.

Serial no.	Name of the Primers	Sequence of the primers
1	PHB-F	GGACTCCTTTCTATAGCAGAGGAGG
2	PHB -R	AAAGTTTGAAGAAGGTGGCCCAG
3	PHV -F	TTGCGGAGGAGACCTTGGCG
4	PHV-R	GATAGTACCACCATTTCCAGTG
5	ATHB8-F	AACACCACTTGACCCCTCAACATCAG
6	ATHB8-R	CACGCAACCAACAAGGCTTATCC
7	ATHB15-F	ATTGGCATCTCAAAATCCTCAGAGA
8	ATHB15-R	GGCAACACGTTCATAACTTCAACAGC
9	ARF10- F	TGGCGAGTCCATGTGTTATC
10	ARF10 - R	TGTAACTTGTTGTTACCGGTGT
11	ARF16 -F	TCAAGCCCGTTAAGCTCTGT
12	ARF16 -R	GGTGGTCTATTCAAGTAGTAATGGTG
13	ARF17-F	CCTCAAAGTGGACTGTCTAGTGC
14	ARF17 -R	GGTGAATAGCTGGGGAGGAT
15	ARF 2-F	TCCTGAGGCTAATCAAGACGA
16	ARF 2-R	GGAGGCTGTCGAGACATATC
17	ARF 3-F	CAACACTTGTTCGGATGGTG
18	ARF 3-R	CCCACACCAAATGTTCCTCT
19	ARF 4-F	ATACTACCCCACCCGGAAAC
20	ARF 4-R	TGAGACTGCATCGCAAAATC
21	SPL3 -F	GGAAAAGCACAACTGACTAAAG
22	SPL3 -R	TGTCGTAGGTTTAGCAGATAGC
23	SPL9 -F	CACTAAACTAGAGAGCTACAAGGG
24	SPL9 -R	GAAGAAGCTCGCCATGTAT
25	SPL10 -F	CATATTTGCTGATGGATTGG
26	SPL10 -R	GTGTTTGATCCCTTGTGAATC
27	AP2-F	TCGACGAACCAAGTGTTGAC
28	AP2-R	CAGCCAATTTTGATGAGGAGT
29	TOE1-F	AAACAAGCGCCGTGAGAC

30	TOE1-R	TAACCACGTGTTGCATTGTC
31	TOE2-F	CATGATCAGTCCAGTGGTG
32	TOE2-R	CTGATTCAGATTGACGAAGG
33	TOE3-F	TAGCAATGATGACGACGACT
34	TOE3-R	GATGATGAATCAGGGACGAG
35	NAC1-F	ACTTTGACCAAGAACCCTCTTC
36	NAC1-R	CTGAGTTGGTTAGGTTCGAGTT
37	CUC1-F	GCAATTGCTCCGATCATCAATAC
38	CUC1-R	GAGCGGGAAGGAATGTATGAA
39	CUC2-F	ACCAACACAACCGTCACA
40	CUC2-R	AGTTAACGTCTAAGCCCAAGG
41	NF-YA8-F	CGGTGGAATCTGGTAATGTGT
42	NF-YA8-R	CTGGACAAAGCGTGTGAAATAAG
43	NF-YA5-F	TCCGGAGACTGGTGATGATA
44	NF-YA5-R	GAGATTGTAGAGGATATGAAAGCAAAG
45	PPR-F	GCTACTCGAATGGTAAGCTGTAT
46	PPR-R	CCTCTGAAACCCTTCCTTCTTT
47	PHO2-F	AAGTGAAGTTTCTCCCGTTAGG
48	PHO2-R	GGAACCCAAGATGTGATTGGA
49	AGL16-F	AGAGGTCGAGGGTGTGAAA
50	AGL16-R	GGACATGTTCGTTCGAGGTATC
51	CIP4.1-F	GTGAGTTGACATCTACTCCAGTTAC
52	CIP4.1-R	GTGCTCCGTTTATCTCGTTCA
53	R3H-F	TTCAACAGCCCTAGCAGTTC
54	R3H-R	CCACCACTTCAGTCTCATTTCT

Supplementary Table S3. The candidate validated targets of known miRNAs during *Arabidopsis* seed germination.

Serial no.	miRNA	Target gene no.(Locus ID)	Description of the target gene	Target annotation	Validated by
1	165/166	AT2G34710	Homeobox-leucine zipper protein PHB	PHB	qRT-PCR
2		AT1G30490	Homeobox-leucine zipper protein PHV	PHV	qRT-PCR
3		AT4G32880	Homeobox-leucine zipper protein ATHB-8	ATHB8	qRT-PCR
4		AT1G52150	Homeobox-leucine zipper protein ATHB-15	ATHB15	qRT-PCR
5	172	AT4G36920	APETALA2	AP2	qRT-PCR
6		AT2G28550	Target of early activation	TOE1	qRT-PCR

			tagged(EAT)1		
7		AT5G60120	Target of early activation tagged(EAT)2	TOE2	qRT-PCR
8		AT5G67180	Target of early activation tagged(EAT)3	TOE3	qRT-PCR
9	390	AT5G62000	Auxin response factor 2	ARF2	qRT-PCR
10		AT2G33860	Auxin response factor 3	ARF3	qRT-PCR
11		AT5G60450	Auxin response factor 4	ARF4	qRT-PCR
12	160	AT2G28350	Auxin response factor 10	ARF10	qRT-PCR
		AT4G30080	Auxin response factor 16	ARF16	qRT-PCR
13		AT1G77850	Auxin response factor 17	ARF17	qRT-PCR
14	156/157	AT2G33810	Squamosa promoter binding protein-like 3	SPL3	qRT-PCR
15	156/157	AT2G42200	Squamosa promoter binding protein-like 9	SPL9	qRT-PCR
16		AT1G27370	Squamosa promoter binding protein-like 10	SPL10	qRT-PCR
17	164	AT3G15170	Cup-shaped cotyledon1(ANAC054, Arabidopsis NAC domain containing protein 54).	CUC1	qRT-PCR
18		AT5G53950	Cup-shaped cotyledon2(ANAC098, Arabidopsis NAC domain containing protein 98).	CUC2	qRT-PCR
19		AT1G56010	NAC domain proteins(ANAC022, Arabidopsis NAC domain containing protein 22).	NAC1	qRT-PCR
20	169	AT1G17590	Nuclear factor Y, subunit A8	NF-YA8	qRT-PCR
21		AT1G54160	Nuclear factor Y, subunit A5	NF-YA5	qRT-PCR
22	161	AT5G16640	Pentatricopeptide repeat (PPR) superfamily protein	(PPR) superfamily protein	qRT-PCR
23	399	AT2G33770	Phosphate 2	PHO2	qRT-PCR
24	824	AT3G57230	AGAMOUS-like 16	AGL16	qRT-PCR
25	834	AT4G00930	COP1-interacting protein 4.1	CIP4.1	qRT-PCR
26	854	AT5G05100	Single-stranded nucleic acid binding R3H protein	R3H	qRT-PCR
